# Multi-omics analysis reveals interactions between host and microbes in Bama miniature pigs during weaning

**DOI:** 10.3389/fmicb.2024.1482925

**Published:** 2024-12-11

**Authors:** Wen Ma, Li Yin, Ying Hu, Xu Liu, Zhenghong Guo, Bingyang Zhong, Haofeng Qiu, Jing Li

**Affiliations:** ^1^Sichuan Key Laboratory of Conservation Biology on Endangered Wildlife, College of Life Sciences, Sichuan University, Chengdu, China; ^2^Chengdu Dossy Experimental Animal Co., Ltd., Chengdu, China; ^3^Dossy Biological Engineering (Chongqing) Co., Ltd., Chongqing, China

**Keywords:** weaning, multi-omics, interactions, gut microbes, metabolites, gene expression

## Abstract

**Introduction:**

There are complex interactions between host and gut microbes during weaning, many of the mechanisms are not yet fully understood. Previous research mainly focuses on commercial pigs, whereas limited information has been known about the host and gut microbe interactions in miniature pigs.

**Methods:**

To address the issue in Bama miniature piglets that were weaned 30 days after birth, we collected samples on days 25 and 36 for metabolomics, transcriptomics, and microgenomics analysis.

**Results and discussion:**

The average daily weight gain of piglets during weaning was only 58.1% and 40.6% of that during 0-25 days and 36-60 days. Metabolomic results identified 61 significantly different metabolites (SDMs), of which, the most significantly increased and decreased SDMs after weaning were ectoine and taurocholate, respectively, indicating the occurrence of inflammation. Metagenomic analysis identified 30 significantly different microbes before and after weaning. Bacteria related to decreasing intestinal inflammation, such as Megasphaera, Alistipes and Bifidobacterium, were enriched before weaning. While bacteria related to infection such as Chlamydia, Clostridium, Clostridioides, and Blautia were enriched after weaning. The carbohydrate enzymes CBM91, CBM13, GH51_1, and GH94 increase after weaning, which may contribute to the digestion of complex plant fibers. Furthermore, we found the composition of antibiotic resistance genes (ARGs) changed during weaning. Transcriptomic analysis identified 147 significantly differentially expressed genes (DEGs). The upregulated genes after weaning were enriched in immune response categories, whereas downregulated genes were enriched in protein degradation. Combining multi-omics data, we identified significant positive correlations between gene MZB1, genera Alistipes and metabolite stachydrine, which involve anti-inflammatory functions. The reduced abundance of bacteria Dialister after weaning had strong correlations with the decreased 2-AGPE metabolite and the downregulated expression of RHBDF1 gene. Altogether, the multi-omics study reflects dietary changes and gut inflammation during weaning, highlighting complex interactions between gut microbes, host genes and metabolites.”

## 1 Introduction

Weaning represents a pivotal stage in mammalian development, characterized by complex metabolic and physiological transformations, including the maturation of the digestive system, alterations in immune function, and restructuring of the gut microbiome (Dogra et al., 2021; Modina et al., 2021; Ames et al., 2023). Nutritional and environmental conditions during this period can exert profound and long-lasting effects on health, both in animals and humans. A thorough understanding of these early influences is crucial for the development of preventive health strategies and interventions aimed at mitigating chronic diseases (Moeser et al., 2017). Importantly, weaning is also associated with an increased risk of diseases such as diarrhea, which can have severe consequences for piglets, including high mortality rates and diminished growth performance. Understanding the nutritional requirements and physiological status during weaning is thus essential for optimizing feeding strategies, ultimately enhancing survival and growth efficiency.

The gut microbiome, a key determinant of host health and disease, plays a significant role in nutrient absorption, immune regulation, and pathogen defense, influenced by shifts in microbial composition and function (Pickard et al., 2017). Weaning, a critical developmental milestone in the mammalian lifecycle, involves a significant dietary transition from liquid milk to solid food, leading to substantial alterations in the gut microbiome composition (Rodríguez et al., 2015). During breastfeeding, milk oligosaccharides and host-derived glycans act as unique carbon sources for the gut microbiome(Mach et al., 2015), while early colonizing gut bacteria facilitate the host’s adaptation to complex carbohydrates after weaning (Guevarra et al., 2018). Weaning is also associated with increased susceptibility to diarrhea, with studies revealing an enrichment of *Enterobacteriaceae* prior to its onset, indicating potential microbial biomarkers for predicting diarrheal risk (Loh et al., 2017). From birth to about 20 days, gut microbes fluctuate significantly (Patil et al., 2019; Wang et al., 2019), and this fluctuation can occur again, more briefly and dramatically after weaning (De Rodas et al., 2018). While the gut microbiota changes within a week after weaning, the initial changes in the microbial community after weaning are quite dramatic(Wei et al., 2017; De Rodas et al., 2018; Patil et al., 2019; Wang et al., 2019; Wang C. et al., 2021). A stable composition of the gut flora will appear at 14 weeks of age (O’Mahony et al., 2005) or even longer at 6 months (Kim et al., 2011). Investigating these short-term shifts can enhance our understanding of how gut microbes rapidly respond to dietary changes, providing a basis for early interventions and improving their effectiveness. Furthermore, after weaning, microorganisms that are better adapted to a high-fiber diet proliferate, while some microbes become linked to intestinal infections and inflammatory responses.

The gut microbiome undergoes profound transformations during weaning, impacting the host significantly. Complex interactions exist between gut microbes, host metabolites and genes. Recent advancements in metagenomics, metabolomics, and transcriptomics have offered new insights into these intricate mechanisms, making this an area of intense research interest. Studies have demonstrated an increase in microbial diversity after weaning (Massacci et al., 2020), with changes in the predominant bacterial populations closely correlating with dietary shifts (Mach et al., 2015; Guevarra et al., 2018). Certain bacterial taxa, notably *Enterobacteriaceae*, are found to proliferate post-weaning and are strongly associated with intestinal inflammation (Loh et al., 2017; Wang et al., 2019). Weaning also significantly alters the metabolic profile of pigs, with notable changes in amino acid, bile acid, and lipid metabolism (Zhao et al., 2018). After weaning, the expression of inflammation-related genes and immune response genes is upregulated, suggesting an activation of the immune system during this critical period (Tang et al., 2022). Although numerous studies have explored the biological changes in weaning piglets, the mechanisms governing host-microbe-metabolite interactions remain inadequately understood, particularly when relying on individual omics datasets.

Extensive researches have investigated the effects of weaning on gut microbiota changes (Massacci et al., 2020), gene expression regulation (Tang et al., 2022), and the complex interactions between gut microbiota and the host (Patil et al., 2019). However, the majority of these studies have been centered on commercial meat pigs. Compared to commercial meat pigs, miniature pigs exhibit significant differences in growth rate, developmental cycle, reproduction, and gene expression (Wang et al., 2017; Shang et al., 2018; Koo and Lee, 2021). For instance, Koo and Lee (2021) demonstrated that miniature pigs have a slower growth rate and a shorter gestation period. Notable differences in gut microbiota composition and metabolic profiles have also been documented across different pig breeds (Patil et al., 2019). Weaning represents a critical phase in the growth and development of pigs, and the associated alterations in gut microbiota and metabolites may differ substantially in Bama miniature pigs compared to those observed in commercial meat pigs. Bama miniature pigs are a unique Chinese breed, characterized by their small body size and early sexual maturity, and they are the most widely utilized pig breed for biomedical research in China, understanding the weaning stress on the gut microbiota and host metabolites and gene expression is critical to the health development of the Bama miniature pigs industry. Meanwhile, with the development of omics technologies, multi-omics approaches offer many advantages in elucidating complex relationships between gut microbiota and the host during weaning. Studies have confirmed that multi-omics approaches enable a systematic exploration of complex biological processes and mechanisms with comprehensiveness, reliability, and personalization (Zhong et al., 2024). In this study, we utilizes a multi-omics approach, integrating metagenomic, metabolomic, and transcriptomic analyses to investigate the mechanistic interactions between gut microbiota and the host in the same 12 Bama miniature pigs, both before and after weaning. This approach will facilitate a comprehensive understanding of the changes occurring during weaning, thereby advancing our knowledge of weaning-related physiological transitions in this important biomedical model.

## 2 Materials and methods

### 2.1 Sample collection

Bama miniature pigs were sourced from Chengdu Dossy Experimental Animal Co., Ltd. Twelve piglets were from six different litters, with each litter one male and one female piglet. The piglets raised with their mothers and were nursed until weaning on day 30 after birth. All piglets received the same diet and housing conditions, and they stayed in the same pens after weaning.

The piglets are primarily breastfed before weaning, supplemented with creep feed (comprising extruded corn, soybean meal, fish meal, whey powder, dicalcium phosphate, limestone powder, sodium chloride, vitamins, pantothenic acid, etc.). The creep feed was provided twice daily at 50 g per litter for piglets. After weaning, each piglet was fed 50 g of creep feed daily for the first 3 days. From day 4 to day 7 after weaning, each piglet was fed 50 g of creep feed and 25 g of piglet compound feed type I (comprising corn, soybean meal, corn germ meal, wheat flour, bran, dicalcium phosphate, limestone powder, sodium chloride, vitamins, etc.).

Weight of the 12 piglets were recorded at 0, 5, 25, 36 and 60 days after birth. Blood and fresh fecal samples were collected on days 25 and 36. Fecal samples were collected using sterile instruments within 10 min of defecation. Samples were directly picked up and stripped of the outer layer that came into contact with the collection tray, stored in 50 mL sterile tubes, frozen, and transported to the laboratory, then stored at −80°C for subsequent metagenomic analysis. Fresh blood samples were collected by professionals and stored in PAXgene blood RNA tubes. They were initially stored at −20°C for about 2 h then transferred to a − 80°C freezer for subsequent transcriptomic analysis. Serum samples were collected in coagulation-promoting tubes, gently inverted 4–5 times immediately after collection to mix the specimens, left at room temperature for 30–60 min, then centrifuged at 3000 rpm for 10 min. The serum was transferred to 1.5 mL centrifuge tubes and stored at −80°C for subsequent metabolomic analysis.

To reduce bias caused by individual difference, this study tracked changes in the same 12 Bama miniature piglets before and after weaning, and collected totaling 72 samples for the sequencing analysis. This finally generated 68 usable datasets for following comparison. In total, our study integrated results from 23 transcriptome data, 24 metabolome data and 21 metagenome data for the following analysis. The dataset includes 24 serum metabolomic samples, 23 blood transcriptomic samples, and 21 fecal metagenomic samples. Three fecal samples (one from 25-day group and two from 36-day group) were removed for metagenomic analysis and one blood sample (from 25-day group) were removed for transcriptomic analysis due to contamination, library preparation or sequencing (Supplementary Table S1). Corresponding study sequences diligently archived in the Sequence Read Archive (SRA) of the National Center for Biotechnology Information (NCBI) website,^1^ accessible under the BioProject designation PRJNA1147800.

This study was approved by the Ethics Committee of the College of Life Sciences, Sichuan University (No. SCU240521001). All sample collection and utility protocols were carried out in strict adherence to the guidelines of the Management Committee of Experimental Animals of Sichuan Province, China (SYXKSichuan, 2019–192).

### 2.2 Metabolomics analysis

#### 2.2.1 Sample extraction and UPLC-Q-TOF analysis

After slow thawing at 4°C, an appropriate volume of the sample was mixed with pre-chilled methanol/acetonitrile/water solution (2:2:1, v/v), vortexed, ultrasonicated at low temperature for 30 min, left at −20°C for 10 min, and then centrifuged at 14,000 g at 4°C for 20 min. The supernatant was vacuum-dried and reconstituted in 100 μL of acetonitrile-water solution (acetonitrile:water = 1:1, v/v) for mass spectrometry analysis. After vortexing, it was centrifuged at 14,000 g at 4°C for 15 min, and the clear supernatant was used for sample analysis. All samples were mixed to prepare QC (Quality Control) samples.

Samples were separated by Ultra-High Performance Liquid Chromatography (UHPLC, Agilent 1,290 Infinity LC). Throughout the analysis, samples were kept in an autosampler at 4°C. To minimize the impact of instrument detection signal fluctuations, samples were analyzed in a randomized order. QC samples were inserted into the sample queue to monitor and assess system stability and data reliability. After separation by the Agilent 1,290 Infinity LC system, samples were analyzed using a Triple TOF 6600 mass spectrometer (AB SCIEX) to collect primary and secondary spectra, using electrospray ionization (ESI) in both positive and negative ion modes.

#### 2.2.2 Data processing

Raw data were converted to mzXML format using ProteoWizard and then processed with XCMS software for peak alignment, retention time correction, and peak area extraction. Data extracted by XCMS underwent metabolite structure identification and data preprocessing before evaluating experimental data quality and proceeding to data analysis.

The processed data were imported into SIMCA-P software for further analysis (version 14.1, Umetric, Umea, Sweden). After being log10 transformed, the peak intensity data were Par scaled. Differential metabolites were screened using both univariate and multivariate statistical analyses. Univariate statistical analysis visualized metabolites with significant fold changes (FC > 1.5 or FC < 0.67, *p <* 0.05) using volcano plots. Multivariate statistical analysis primarily involved orthogonal partial least squares discriminant analysis (OPLS-DA). The model’s fit was evaluated through 200 permutation tests. Potential biomarkers between groups were identified using Variable Importance in Projection (VIP) scores derived from OPLS-DA, with significant differential metabolites (SDMs) selected based on VIP > 1 and *p <* 0.05. Identified metabolites were mapped onto the Kyoto Encyclopedia of Genes and Genomes (KEGG) database using KEGG mapper (Kanehisa, 2017). KEGG pathway enrichment analysis of differential metabolites was performed using MSEA (Metabolite Sets Enrichment Analysis) (Deng et al., 2021), and a Venn diagram was drawn using the web application: http://jvenn.toulouse.inra.fr/app/index.html.

#### 2.2.3 Weighted gene co-expression network analysis

A co-expression network for the metabolite peak intensity matrix was constructed using the R package WGCNA v1.721 (Zhang and Horvath, 2005; Langfelder and Horvath, 2008). An initial similarity matrix was obtained through Pearson correlation. The appropriate soft-thresholding power (*β*) for network construction was determined using the “pickSoftThreshold” function within the WGCNA package. The “blockwiseModules” function was used to construct gene co-expression networks and detect genomic modules. The “moduleEigengenes” function calculated the module eigengenes (ME), representing the first principal component of gene expression for a given module. Finally, the Pearson correlation coefficients between the module (ME) and traits (stages), between each gene and the module’s expression levels [module membership (MM)], and between each gene and the traits’ expression levels [gene significance (GS)] were calculated. This analysis generated a correlation matrix for all metabolites with stages, and metabolites significantly related to stages were identified using a correlation coefficient criterion of |r| > 0.5 and *p <* 0.05. Hub metabolites were selected based on gene significance (GS) and module membership (MM), with criteria of MM > 0.8 and GS > 0.2.

### 2.3 Metagenomics analysis

#### 2.3.1 Sample extraction, library preparation and sequencing

Total DNA from fecal samples was extracted using the Tiangen DNA Stool Magnetic Bead Kit (Tiangen Biotech, China) according to the manufacturer’s protocol. DNA purity and integrity were assessed using 1% agarose gel electrophoresis (AGE). DNA quantification was performed using the Qubit® dsDNA Assay Kit on a Qubit® 2.0 Fluorometer (Life Technologies, CA, United States).

Fecal samples underwent metagenome sequencing at Novogene (Beijing, China) on the Illumina NovaSeq 6,000 platform. For library construction, 1 μg of genomic DNA was used for library construction with the NEBNext® Ultra DNA Library Prep Kit for Illumina (NEB, USA). Then the initial quantification was conducted with Qubit 2.0, followed by insert size verification using the Agilent 2,100. After confirming the expected insert size, the effective concentration of the library was accurately quantified using Quantitative Real Time Polymerase Chain Reaction (Q-PCR) (library effective concentration > 3 nM) to ensure library quality. Sequencing was performed using the Illumina platform, with a paired-end sequencing length of 150 bp (PE150).

#### 2.3.2 Data analysis

Quality control and identification. Following the acquisition of raw metagenome data, adapters and low-quality reads were removed using Trimmomatic v0.39 (Bolger et al., 2014). The Bama miniature pig reference genome was downloaded from NCBI (*S. scrofa*, Sscrofa11.1). High-quality reads processed were aligned to this reference genome, and potential host sequences were removed using Bowtie2 v2.5.1 (Langmead and Salzberg, 2012). The quality of the metagenomes was assessed using the FastQC v0.11.9. The microbial taxa in the fecal metagenomes were identified using Kraken2 v2.0.7 (Wood and Salzberg, 2014). The results of Kraken2 were corrected using Bracken v2.9 (Lu et al., 2017) to recalculate the actual abundance of each classification unit based on the classification distribution and length distribution information output by Kraken 2.

Gene assembly, functional prediction, and quantification. Metagenomes were assembled using MEGAHIT v1.2.9 to obtain contigs longer than 300 bp (Li et al., 2015). Open reading frames (ORFs) were predicted from the assembled contigs using Prodigal v2.6.3 (Hyatt et al., 2010). A non-redundant gene set was constructed using CD-HIT v4.8.1 (Fu et al., 2012) with a similarity threshold of 95% and coverage over 90%. The genes were translated into amino acid sequences using Biopython. Functional annotation was performed by aligning the amino acid sequences against protein databases from the Carbohydrate-Active enZYmes Database^2^ and the Comprehensive Antibiotic Resistance Database^3^ using Diamond v2.1.8.162.

Gene family abundances and microbial metabolic pathways were quantified using HUMAnN 3.0 v3.8 based on the ChocoPhlAn database (chocophlan_v296_20190) and the UniRef90 database (uniref90_v201901) (Suzek et al., 2007; Beghini et al., 2021).

Differences in microbial abundance before and after weaning were determined using LEfSe v1.1.2 (Linear Discriminant Analysis, LDA > 3, *p <* 0.05; Segata et al., 2011). Significant differences in CAZymes, Antibiotic resistance genes, and functional pathways before and after weaning were identified using the Wilcoxon test with False Discovery Rate (FDR) for multiple testing corrections (*p <* 0.05 for CAZymes and ARGs, *p <* 0.01 for functional pathways).

### 2.4 Transcriptomics analysis

#### 2.4.1 Sample extraction, library preparation and sequencing

Total RNA was extracted from blood samples using the PAXgene Blood RNA Kit, according to the manufacturer’s instructions. RNA quality was assessed using an Agilent 4,200 Bioanalyzer (Agilent Technologies, Santa Clara, CA, United States). Preliminary quantification was performed using a NanoDrop 2000 spectrophotometer, followed by precise concentration measurement with the Agilent 4,200. Blood samples with an RNA Integrity Number (RIN) greater than 6.0 were used for subsequent library construction and sequencing (sample BM12 36d had a RIN of 4.4 and was riskily processed for library construction).Transcriptome sequencing of blood samples was performed at Berry Genomics (Beijing, China) using the Illumina NovaSeq 6,000 platform.

#### 2.4.2 Data analysis

Quality control and reads alignment. The quality of raw sequencing data was assessed using FastQC v0.12.1. The NGSQC Toolkit v2.3 was used to remove adapters and low-quality reads based on stringent criteria: removing sequences with adapters, sequences with more than 5% N bases, and sequences where more than 70% of bases with a quality score ≤ 20 were discarded (Liu et al., 2012). Subsequently, a genome index was constructed for Sscrofa11.1, and high-quality reads were mapped to the reference genome using HISAT2 v2.1.0 (Kim et al., 2015). SAM files were converted to BAM files using SAMtools v1.19.2, and sorted.bam files were organized for subsequent assembly. Finally, raw read count matrices for each gene and transcript were obtained using featureCounts v2.0.6 (Liao et al., 2014), and low-expression genes were removed.

High expression gene screening. Fragments Per Kilobase of exon model per Million mapped fragments (FPKM) and Transcripts Per Million (TPM) were calculated to normalize raw count data in each sample. Genes with a TPM ≥ 20.0 were selected as criteria for high-expression genes. A gene with a TPM value ≥20.0 in both before-weaning and after-weaning samples was identified as a commonly high-expressed gene. A gene expressed with a TPM ≥ 20.0 only in the before-weaning or after-weaning phase was identified as a stage-specific high-expressed gene.

Differentially expressed gene screening. Differential expression analysis was conducted using the R package edgeR v4.0.3 (Robinson et al., 2010). The fold change (FC) was used to screen for differentially expressed genes (DEGs), and genes meeting the criteria of |log_2_FC| ≥ 1.5 and an FDR ≤ 0.05 were considered significantly differentially expressed.

Weighted gene co-expression network analysis (WGCNA) and hub gene selection. A co-expression network was constructed using the R package WGCNA. The method was consistent with the metabolome analysis. Hub genes were selected based on gene significance (GS) and module membership (MM) (MM > 0.8 and GS > 0.2).

Gene function enrichment analysis. Gene Ontology (GO) and KEGG pathway enrichment analyses were performed using g: Profiler to calculate the gene ratio to explore the biological functions of stage-specific highly expressed genes, DEGs, and hub genes (Raudvere et al., 2019). The analysis used *Sus scrofa* as the background species.

### 2.5 Correlation analysis

The correlation between microbes, genes, and metabolites was calculated using the R package psych v2.4.6.26, and visualization was performed with the R package ggplot2 v3.5.0. Cytoscape v3.10.1 was used to visualize interactions with a correlation coefficient greater than 0.8.

## 3 Results

### 3.1 Weight changes of piglets during weaning

We recorded the weight changes in the 12 Bama miniature piglets from birth to 60 days. The statics results showed that during the weaning period (25–36 days), the piglets had an average daily weight gain of 0.054 ± 0.015 kg. It was only 58.1 and 40.6% of the average daily weight gain in piglets of 0–25 days and 36–60 days, indicating a significant slowdown in growth rate during the weaning (Table 1).

**Table 1:** Changes in weight of the 12 piglets from 0 to 60 days after birth.

Time (after weaning)	0d	25d	36d	60d
Weight (average value/kg)	0.695 ± 0.136	3.021 ± 0.706	3.617 ± 0.868	6.804 ± 1.649
Average daily weight gain (/kg)	–	0.093 ± 0.023	0.054 ± 0.015	0.133 ± 0.033

### 3.2 Changes on serum metabolites in piglets during weaning

**Figure 1:**
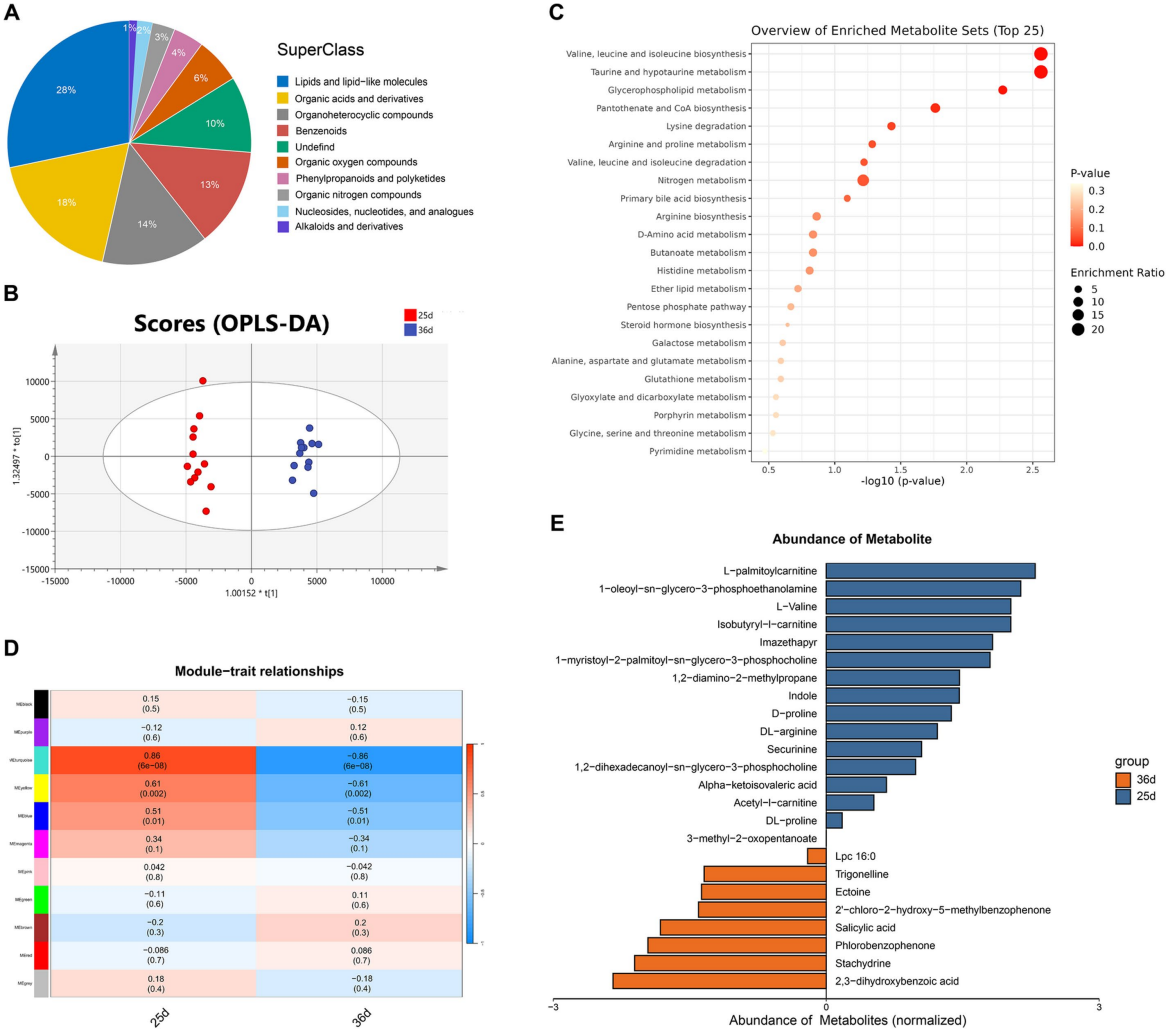
Metabolomic analysis of Bama miniature pigs before and after weaning. **(A)** Metabolite superclass. **(B)** OPLS-DA score plots of serum metabolites before and after weaning. **(C)** KEGG enrichment results of all DEMs **(D)** Correlation heatmap between modules and different stages (metabolites). **(E)** Abundance of hub metabolites before and after weaning.

UPLC-Q-TOF-based metabolomics analysis of serum from the 12 piglets identified 660 metabolites in positive ion mode and 608 in negative ion mode (Supplementary Table S2). In total, the 1,268 metabolites primarily consisted of lipids and lipid-like molecules (27.8%), organic nitrogen compounds (18.4%), organic acids and their derivatives (14.4%), and benzenoids (12.9%) (Figure 1A). KEGG annotations indicated that these metabolites are primarily involved in pathways associated with amino acid metabolism (e.g., valine, leucine, and isoleucine biosynthesis), lipid metabolism (e.g., primary bile acid biosynthesis), among others (Supplementary Figure S1A). Regarding relative abundance, there was a notable decrease in lipids and lipid-like molecules, organic nitrogen compounds, and organic acids and their derivatives, whereas benzenoids and organic oxygen compounds exhibited an increase after weaning (Table 2).

**Table 2:** Relative abundance of metabolite superclass in piglets.

Metabolite	25d	36d	Trend
Organic oxygen compounds	5.66%	6.86%	Up
Benzenoids	5.45%	6.65%	
Phenylpropanoids and polyketides	0.69%	0.73%	
Organosulfur compounds	0.06%	0.07%	
Alkaloids and derivatives	0.05%	0.21%	
Organic acids and derivatives	41.67%	40.67%	Down
Lipids and lipid-like molecules	28.00%	26.75%	
Organic nitrogen compounds	6.16%	5.89%	
Organoheterocyclic compounds	5.91%	5.48%	
Nucleosides, nucleotides, and analogs	0.54%	0.45%	

#### 3.2.1 Differential metabolite before and after weaning

Supervised OPLS-DA revealed a clear distinction between metabolites from the two time points (Figure 1B), highlighting significant differences in the metabolic profiles of Bama miniature piglets before and after weaning. Differential analysis of all metabolites was visualized using volcano plots (FC > 1.5 or FC < 0.67, *p* < 0.05; Supplementary Figure S1B). In total, 60 significantly differential metabolites (SDMs) were identified between days 25 and 36 (VIP > 1 and *p* < 0.05). Among these, 16 SDMs were significantly upregulated, while 44 SDMs were significantly downregulated post-weaning. Ectoine (fold change = 7.59) demonstrated the highest increase, whereas taurocholate (fold change = 0.12) showed the most substantial decrease in the piglets following weaning (Supplementary Table S3). Enrichment analysis of the SDMs identified five significant KEGG pathways (*p* < 0.05) that were downregulated after weaning, including valine, leucine, and isoleucine biosynthesis, taurine and hypotaurine metabolism, glycerophospholipid metabolism, pantothenate and CoA biosynthesis, and lysine degradation (Figure 1C).

#### 3.2.2 Hub metabolite screening

WGCNA was employed to identify metabolites correlated with weaning. Metabolites were grouped into 11 modules, with three of these showing significant module-trait correlations with weaning (*p* < 0.05; Figure 1D). The turquoise module (*r* = 0.86, *p* = 6e-8), yellow module (*r* = 0.61, *p* = 0.002), and blue module (*r* = 0.51, *p* = 0.01) exhibited significant positive correlations with the 25-day group and negative correlations with the 36-day group. The turquoise module contained 219 metabolites (including 33 SDMs), the yellow module contained 178 metabolites (including 11 SDMs), and the blue module contained 212 metabolites (including 11 SDMs; Supplementary Table S4).

Combining MM > 0.8 and GS > 0.2 criteria, 82 metabolites were identified across the three modules, of which 24 were also SDMs and positively correlated with the 25-day group (hub metabolites) (Supplementary Table S5). Notably, 16 metabolites, such as isobutyryl-L-carnitine, L-palmitoylcarnitine, acetyl-L-carnitine, and securinine, were more abundant prior to weaning, whereas 8 metabolites, including stachydrine, trigonelline, ectoine, and salicylic acid, were more abundant post-weaning (Figure 1E).

#### 3.2.3 Biomarker screening

Receiver Operating Characteristic (ROC) analysis of hub metabolites identified 15 potential biomarkers (Area Under the Curve, AUC > 0.9), including securinine, isobutyryl-L-carnitine, L-palmitoylcarnitine, DL-arginine, DL-proline, 1,2-diamino-2-methylpropane, stachydrine, trigonelline, ectoine, L-valine, 2,3-dihydroxybenzoic acid, phlorobenzophenone, 2′-chloro-2-hydroxy-5-methylbenzophenone, D-proline, and alpha-ketoisovaleric acid. Among these, 2,3-dihydroxybenzoic acid exhibited the highest predictive accuracy, with an AUC of 0.986 (Supplementary Figure S1C).

### 3.3 Changes on gut microbiota in piglets during weaning

**Figure 2:**
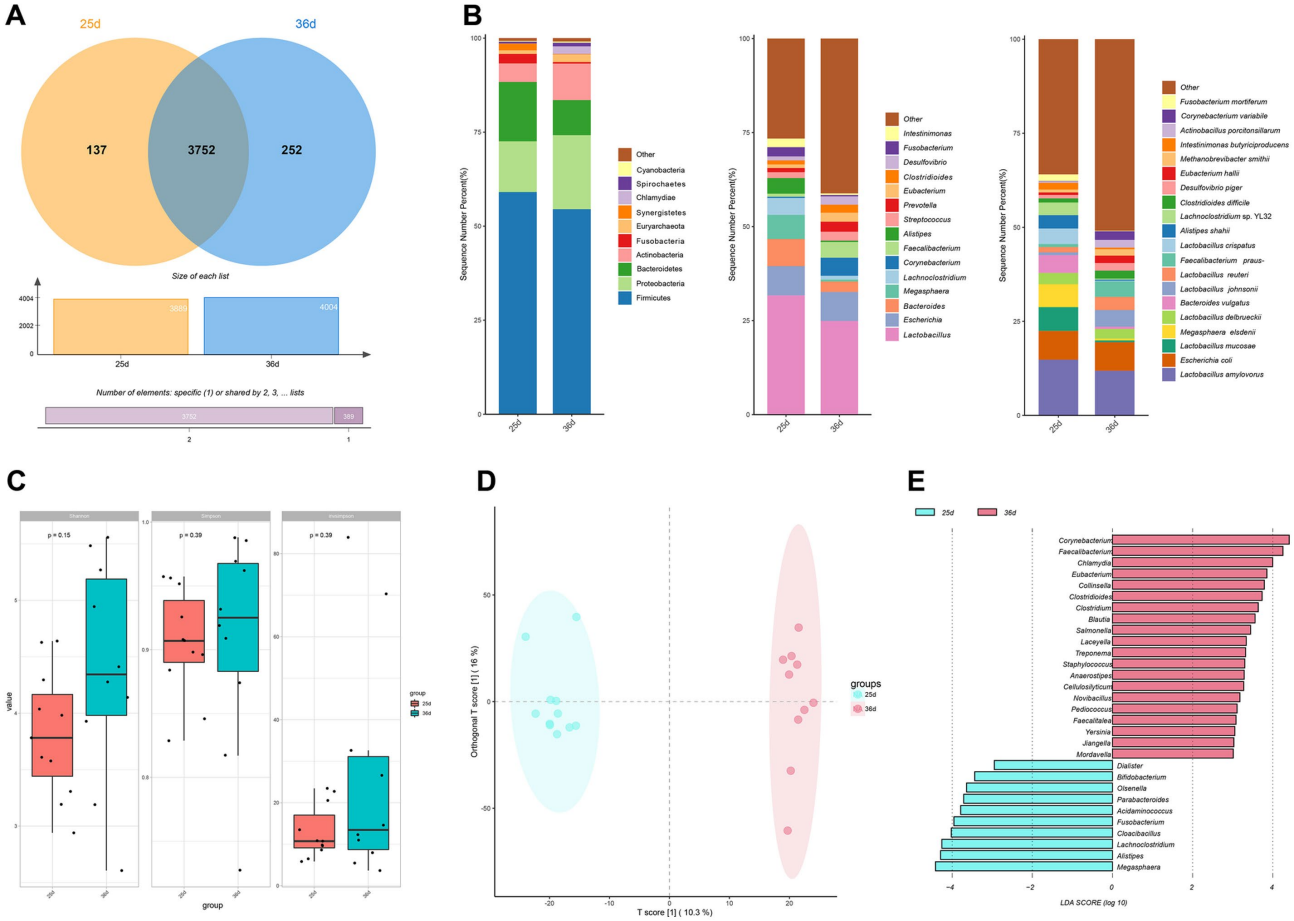
Analysis of the composition of the gut microbiota. **(A)** Venn diagram of the number of species before and after weaning. **(B)** Relative gut microbiota abundance at the phylum, genus, species level before and after weaning. **(C)** Alpha diversity of gut microbiota in piglets before and after weaning. **(D)** OPLS-DA plots of fecal metagenomics before and after weaning. **(E)** LEfSe analysis before and after weaning.

Following quality control, metagenome sequencing generated an average of 43,985,77 high-quality clean read pairs per sample (Supplementary Table S6). Rarefaction curves reached a plateau, confirming sufficient sequencing depth (Supplementary Figures S2A,B). The gut microbiota of piglets comprised 40 phyla, 1,296 genera, and 4,141 species. At the species level, 3,752 microbial species were shared between piglets pre- and post-weaning, while 137 and 252 species were unique to piglets before and after weaning, respectively (Figure 2A). The gut microbiota was primarily dominated by the phyla Firmicutes, Proteobacteria, and Bacteroidetes, with *Lactobacillus* being the predominant genus, and *Lactobacillus amylovorus* the most dominant species (Figure 2B). Diversity analysis revealed a increase in richness (Figure 2C).

#### 3.3.1 Significant differential microbes

OPLS-DA analysis revealed distinct differences in microbiota composition between the two time periods, indicating significant changes in gut microbiota during weaning (Figure 2D). LEfSe analysis identified 30 significantly differential genera between piglets at 25 days and 36 days (LDA > 3, *p* < 0.05; Figure 2E). Genera significantly enriched at 25 days included *Megasphaera*, *Alistipes*, *Lachnoclostridium*, *Fusobacterium*, *Cloacibacillus*, *Acidaminococcus*, *Parabacteroides*, *Olsenella*, *Bifidobacterium*, and *Dialister*. In contrast, genera significantly enriched at 36 days included *Corynebacterium*, *Chlamydia*, *Faecalibacterium*, *Eubacterium*, *Collinsella*, *Clostridium*, *Clostridioides*, *Blautia*, *Salmonella, Laceyella, Treponema*, *Staphylococcus*, *Pediococcus*, *Anaerostipes*, *Staphylococcus*, *Novibacillus*, *Faecalitalea*, *Yersinia*, *Jiangella*, *Mordavella*.

ROC analyses of the significantly different microbes identified *Faecalibacterium*, *Alistipes*, *Dialister*, *Corynebacterium* as several potential biomarkers with AUC values greater than 0.9. Of these, *Faecalibacterium* had the largest AUC value of 0.982 (Supplementary Figure S2C).

#### 3.3.2 Functional changes on microbiota

**Figure 3:**
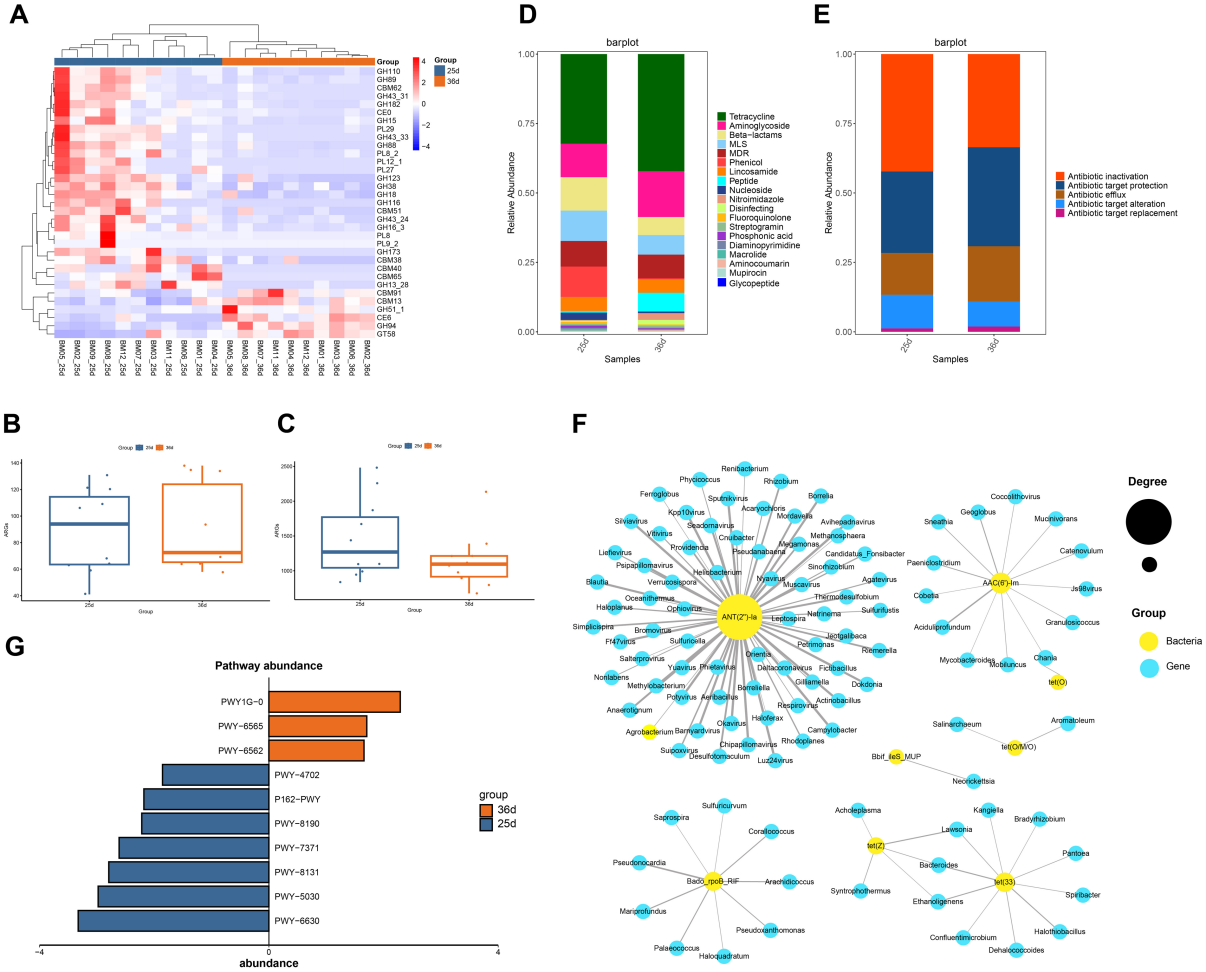
Functional analysis of the gut microbiota. **(A)** Heatmap of relative abundance clustering of carbohydrate enzymes with significant differences before and after weaning. **(B)** Diversity of ARGs before and after weaning. **(C)** Abundence of ARGs before and after weaning. **(D)** Distribution of antibiotic classes. **(E)** The antibiotic resistance mechanisms of the ARGs. **(F)** The abundance of pathway before and after weaning. **(G)** The abundance of pathway before and after weaning.

After assembling the clean data (Supplementary Table S6), CAZyme annotation of gut microbes identified six primary classifications and 382 secondary classifications. Glycoside Hydrolases (GHs) represented the highest number of genes, with GT2, GT4, GH3, GH1, and GH2 being the dominant enzymes within the GH family, mainly involved in carbohydrate and polysaccharide breakdown (Supplementary Figure S3A). Both the diversity and abundance of CAZymes were reduced in gut microbiota after weaning (Supplementary Figures S3B,C). Using Wilcoxon and FDR tests (*p* < 0.05), we identified 33 CAZymes that differed significantly in abundance between the two groups (Figure 3A), suggesting substantial differences in carbohydrate digestion and absorption during weaning. Notably, 6 CAZymes were more abundant post-weaning, including CBM91, CBM13, GH51_1, CE6, GH94, and GT58, which are primarily associated with the digestion of complex fibers.

To identify ARGs in the gut metagenomes of Bama miniature pigs before and after weaning, DIAMOND was used in BLASTP mode (Identity 90%, Query Coverage 90%) to align ORFs with the CARD database. A total of 231 ARGs were identified, and OPLS-DA analysis indicated that samples could be clustered separately before and after weaning (Supplementary Figure S3D). Among them, 178 ARGs were identified in piglets before weaning, while 207 were found after weaning. ARGs diversity was higher after weaning (Supplementary Table S7), although ARGs abundance was lower (Figures 3B,C). These ARGs conferring resistance to 19 antibiotics classes (Figure 3D), belonging to three major resistance mechanisms, antibiotic inactivation, antibiotic target protection, and antibiotic efflux (Figure 3E). Among these, glycopeptide antibiotic resistance was not found before weaning, while resistance to the other 18 antibiotic existed both before and after weaning. Genes related to tetracycline, aminoglycoside, beta-lactam, and macrolide-lincosamide-streptogramin (MLS) were more abundant and widely distributed than other ARGs. Tetracycline resistance was the most abundant, accounting for 32 and 42% of all ARGs in piglets before and after weaning, respectively. After weaning, the abundance of antibiotic resistance genes such as tetracycline, aminoglycoside and peptide increased (Figure 3D).

Using the ARG risk assessment framework by Zhang et al., we categorized the identified ARGs into four risk levels: ARGs unrelated to humans (Rank IV), non-mobile ARGs (Rank III), ARGs with potential future risks but not yet present in pathogens (Rank II), and ARGs already found in pathogens (Rank I)(Zhang et al., 2021). The number of ARGs in each category was 62 (Rank IV), 44 (Rank III), 16 (Rank II), and 19 (Rank I) (Supplementary Table S8). Before weaning, 15.7% of ARGs belonged to Ranks I and II, while after weaning, this proportion increased to 16.4%, indicating elevated ARG risks after weaning(Table 3). Among the six ARGs that increased in Ranks I and II after weaning, five encoded resistance to aminoglycosides, and one to MLS.

**Table 3:** Risk classification at each level of the ARGs.

Risk levels	I	II	III	IV	Notassessed	Total
25d	17	11	38	40	72	178
36d	18	16	38	56	79	207
Total	19	16	44	62	90	231

A total of 154 ARGs were shared by both piglets, with 24 ARGs unique before weaning and 53 unique after weaning in piglets. Nine ARGs showed significant differences before and after weaning (Wilcoxon test and FDR, *p* < 0.01). Specifically, *Bado_rpoB_RIF*, *Bbif_ileS_MUP*, and *ErmQ* were more abundant before weaning, while *AAC(6′)-Im*, *ANT(2″)-Ia*, *tet(33)*, *tet(O)*, *tet(O/M/O)*, and *tet(Z)* were significantly enriched after weaning. The significantly different ARGs after weaning largely conferred resistance to aminoglycosides and tetracyclines.

Network analysis revealed interactions between significantly different ARGs and microbe genus (*p* < 0.01; Figure 3F). *ANT(2″)-Ia* gene showed the highest degree, with significant associations with 68 genera of microbes, primarily from bacteriophage and the phyla Proteobacteria and Bacteroidetes, suggesting that *ANT(2″)-Ia* is a highly mobile resistance gene. Aminoglycoside resistance genes were most influenced by microbial interactions, followed by tetracycline resistance genes. *Lawsonia*, *Bacteroides*, and *Ethanoligenens* were associated with both *tet(Z)* and *tet(33)*, which may indicate that these genera predominantly affect the abundance of tetracycline resistance genes.

Functional profiling of gut bacteria using HUMAnN3 revealed 10 pathways with significant differences between piglets before and after weaning (Wilcoxon test, FDR, *p* < 0.01). Pathways related to energy metabolism, including 1,4-dihydroxy-6-naphthoate biosynthesis II (PWY-7371), superpathway of L-tyrosine biosynthesis (PWY-6630), L-histidine degradation III (PWY-5030), 5,6-dimethylbenzimidazole biosynthesis I (aerobic) (PWY-8131), L-glutamate degradation XI (PWY-8190), L-glutamate degradation V (P162-PWY), and phytate degradation I (PWY-4702), were enriched prior to weaning. In contrast, pathways associated with infection and immune responses, such as norspermidine biosynthesis (PWY-6562), the superpathway of polyamine biosynthesis III (PWY-6565), and mycothiol biosynthesis (PWY1G-0), were enriched following weaning (Figure 3G).

### 3.4 Changes on the gene expression in piglets during weaning

**Figure 4:**
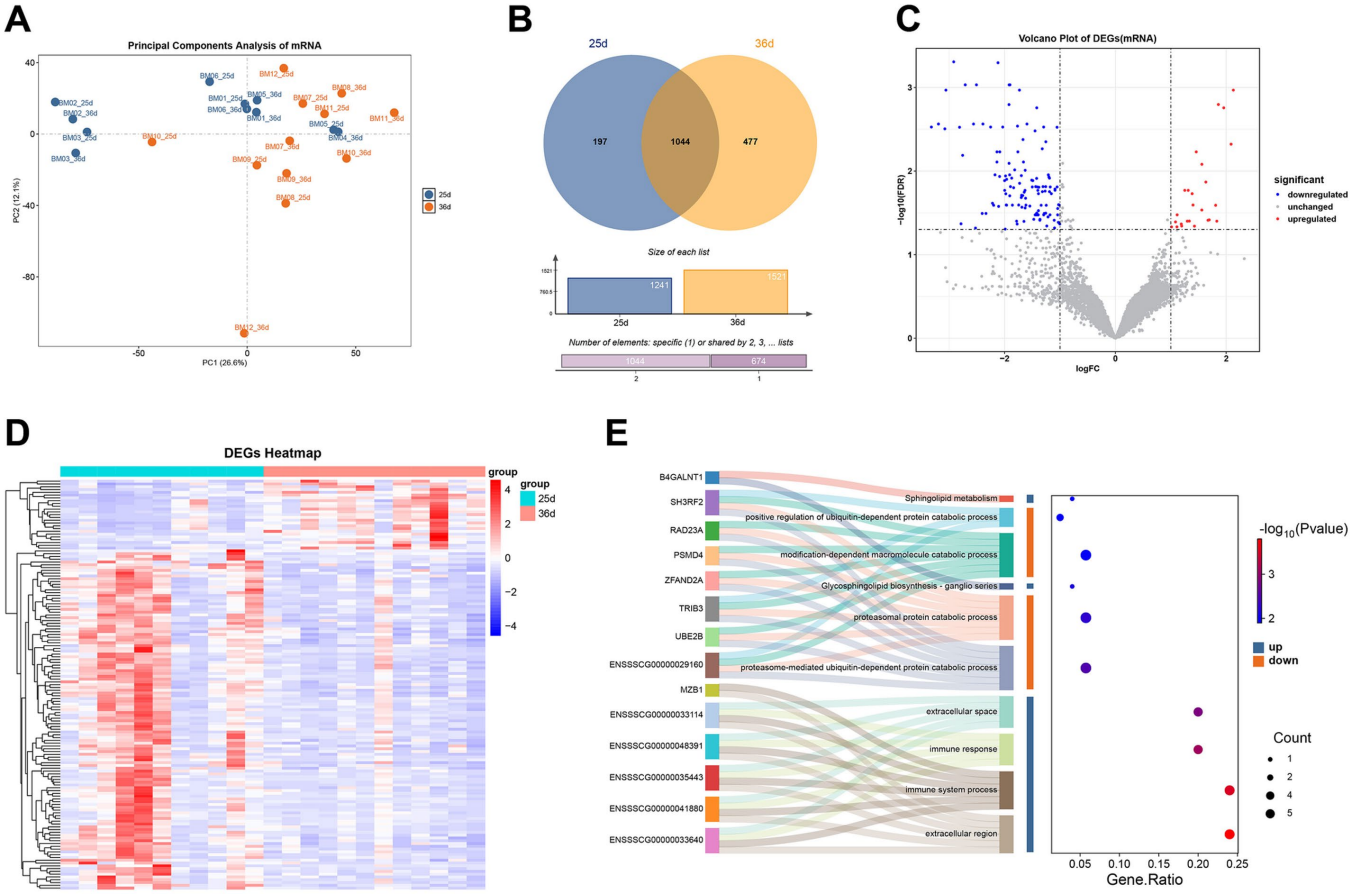
Transcriptomics analysis of Bama miniature pigs before and after weaning. **(A)** PCA plot of gene expression. **(B)** Venn diagrams of highly expressed genes (TPM ≥ 20) before and after weaning. **(C)** Volcano plot of DEGs. **(D)** Heatmap of DEGs. **(E)** Sankey plot of GO and KEGG enrichment results for DEGs (Top 10).

Upon quality control, an average of 29,682,072 high-quality clean read pairs were obtained per sample (Supplementary Table S9). The average alignment rate of clean data to the Sscrofa11.1 reference genome was 97.27% (Supplementary Table S9), confirming the high alignment quality suitable for subsequent analyses. Principal Component Analysis (PCA) revealed a clear distinction in gene expression profiles between piglets before and after weaning (Figure 4A).

#### 3.4.1 The highly expressed genes

Based on TPM expression levels (TPM ≥ 20), shared and specifically highly expressed genes were identified in piglets before and after weaning. A total of 197 and 477 genes were highly expressed specifically at 25 days and 36 days, respectively (Figure 4B). Enrichment analysis indicated that genes highly expressed pre-weaning were enriched in pathways related to intracellular anatomical structures, while those highly expressed post-weaning were enriched in immune system-related pathways (Supplementary Figure S4A).

#### 3.4.2 Significantly differentially expressed genes

A total of 147 DEGs were identified (|logFC| > 1 and FDR < 0.05), with 25 upregulated and 122 downregulated after weaning (Figure 4C). Heatmap showed that the expression of DEGs was different before and after weaning (Figure 4D). A relatively large number of DEGs were down-regulated after weaning. *ENSSSCG00000053814*, *ENSSSCG00000040986*, and *CORO6* were the top three genes that were up-regulated with the highest fold change after weaning, whereas the ones that were down-regulated with the highest fold change were *IL20RB*, *MAFF*, and *TGM5*. Among them, *IL20RB* was related to interleukin 20, and *MAFF* might be involved in cellular stress. Although there were some differences between individuals, the majority of individuals showed the same trend. After weaning, the upregulated DEGs were enriched in immune response-related categories, while downregulated DEGs were enriched in protein degradation categories (Figure 4E), suggesting complex immune responses and inflammatory might occur during weaning.

WGCNA identified genes correlated with weaning, grouping all genes into 35 modules. One module (blue module) was significantly correlated with weaning (Supplementary Figure S4B). This module contained 701 genes and was significantly positively correlated with the 25-day group and negatively correlated with the 36-day group (*R*^2^ = 0.62, *p* = 0.002). Filtering for MM > 0.8 and GS > 0.2, 256 genes were identified, of which 80 were also DEGs, potentially playing key roles during weaning (hub genes). Enrichment analysis of these genes indicated their involvement primarily in protein synthesis and metabolic pathways, with higher expression at 25 days compared to 36 days (Supplementary Table S10).

### 3.5 Correlation analysis on the SDMs, differential microbes, and DEGs

**Figure 5:**
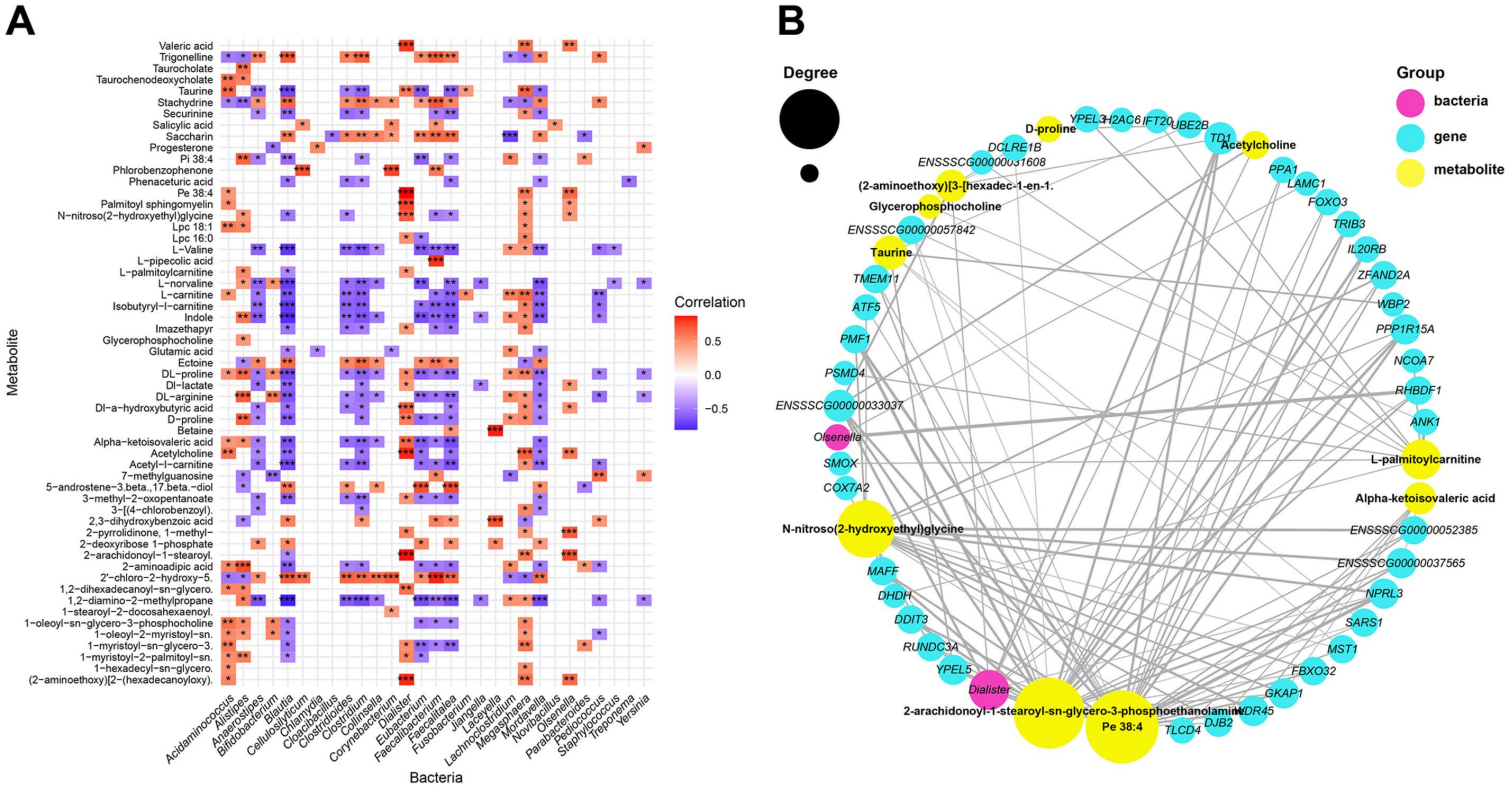
Multi-omics analysis of interactions. **(A)** Heatmap summarizing the correlation of SDMs and significantly different microorganisms (*p < 0.05, **p < 0.01, and ***p < 0.001). **(B)** Network correlation between SDMs, significantly different microorganisms and hub genes.

To explore correlations between SDMs, differential microbes, and DEGs, a correlation matrix was constructed using Spearman’s correlation coefficient. A total of 57 SDMs were significantly correlated with 29 differential microbial genera, resulting in 421 significant correlations (|r| > 0.4, *p* < 0.05), including 198 positive and 223 negative correlations (Figure 5A). Furthermore, 54 SDMs showed significant correlations with 139 DEGs, with 4,147 significant correlations, comprising 3,018 positive and 1,129 negative correlations (Supplementary Table S11). Additionally, 27 differential microbial genera were significantly correlated with 147 DEGs, with 558 significant correlations, including 467 positive and 91 negative correlations (Supplementary Table S12).

Notably, 6 upregulated DEGs in piglets after weaning were related to immune-related biological processes, among which, gene *ENSSSCG00000033640* was significantly positively correlated with bacteria *Alistipes*, *Blautia*, *Pediococcus* and *Acidaminococcus*, and significantly negatively correlated with metabolites acetyl-l-carnitine, DL-arginine and L-carnitine. Similarly, gene *ENSSSCG00000041880* was significantly positively correlated with bacteria of genera *Pediococcus*, and was significantly negatively correlated with metabolites acetyl-l-carnitine. Gene *MZB1* was significantly positively correlated with bacteria of genera *Alistipes* and metabolites stachydrine, indole and trigonelline. These metabolites and microbes were all reported to be related to immune responses.

Network analysis demonstrated correlations between SDMs, differential microbes, and hub genes (correlation coefficient > 0.8, *p* < 0.05; Figure 5B). A total of 113 pairs of highly positively correlated relationships were identified. Pe 38:4, *Dialister*, and *TD1* were the top-ranked SDMs, significantly different microbe, and hub gene, respectively. Pe 38:4 was significantly correlated with 25 hub genes, including *TD1* and *YPEL5*, as well as one significantly differential bacterium, *Dialister*. *Dialister* was significantly correlated with four SDMs, including Acetylcholine and Valeric acid, and five hub genes, including *TD1, NPRL3, PPP1R15A, RHBDF1* and *ENSSSCG00000033037*. Notably, a high correlation was found between the bacteria of the genus *Dialister*, the metabolite 2-AGPE, and the gene *RHBDF1*, all of which are reported to be associated with gut inflammation and they were showed downregulated in piglets after weaning.

## 4 Discussion

Weaning is a critical stage in the growth and development of pigs, during which significant changes occur in the intestinal microbiome, metabolites, and gene expression. These transformations have profound implications for the growth, immune function, and overall health of pigs. This study conducted a longitudinal analysis combining multi-omics data (metabolome, transcriptome, and metagenome) in Bama miniature pigs before and after weaning (25 days and 36 days after birth), providing comprehensive data on the gut microbiota, metabolite profile and gene expression profile during weaning, which will deepen our understandings on the host-microbe-metabolite interactions during weaning.

### 4.1 Multi-omics results indicate dietary change and malnutrition in piglets during weaning

According to feeding records, all 12 piglets were separated from the sow at 30 days of age and subsequently fed exclusively with artificial feed. This dietary transition was reflected in both metabolomic and metagenomic analyses. Metabolomic analysis revealed elevated levels of lipids and lipid-like molecules, as well as organic acids and their derivatives, in piglets prior to weaning. Consistent with our results, López-López et al. found that variations in dietary composition lead to changes in fatty acid content in human infant feces(López-López et al., 2001), likely due to the presence of organic acids, high fat and caloric content in breast milk(González et al., 2023). Similarly, Andreas et al. noted that breast milk is rich in lipids and phospholipids, which are key components of cell membranes (Andreas et al., 2015). In our study, the elevated levels of three carnitine esters and four phospholipids observed in piglets prior to weaning reflect the active metabolic state characteristic of neonates. Furthermore, metagenomic data highlighted dietary changes associated with weaning. Initially, the gut microbiota is relatively sparse at birth, progressively becoming more complex and diverse with dietary shifts. Our study supports this finding, demonstrating an increase in gut microbial diversity following weaning. This is consistent with Wang et al., who reported similar trends in the intestinal microbiome of pigs at various developmental stages (Wang et al., 2019). Bacteria of genera *Bifidobacterium,* known to metabolize milk oligosaccharides, are prevalent in human infants during breastfeeding (Ma et al., 2022). Correspondingly, our study identified a decrease in *Bifidobacterium* levels following weaning. During the breastfeeding phase, oligosaccharides and host-derived glycans serve as unique carbon sources for gut microbes. Following weaning, early colonizing gut bacteria facilitate adaptation to complex carbohydrates (Al Nabhani and Eberl, 2020). In our study, carbohydrate-active enzymes such as CBM91, CBM13, GH51_1, and GH94 were observed to increase in the gut microbiota of piglets post-weaning, aiding in the digestion of complex plant fibers.

During weaning, piglets may experience a brief period of anorexia, which leads to reduced growth rates and intestinal inflammation (Pluske, 2016; Meale et al., 2017; Blavi et al., 2021). Our results on the daily weight gain of piglet were consistent with the conclusion, showing a significant slowdown in growth during weaning compared with that before weaning (0–25 days) and after weaning (36–60 days). Acute stress caused by weaning also could induce significant shifts in metabolite levels. González et al. reported that sudden weaning stress increased the demand and utilization of tyrosine, reducing its blood concentration (González et al., 2023). In our study, hub metabolites, including four amino acids and their derivatives, were decreased in piglets after weaning. Stress caused by weaning might slow protein synthesis while increasing demand and utilization, resulting in lower observed levels. Nie et al. found that branched-chain amino acids stimulate protein synthesis (Nie et al., 2018). Thus, we hypothesize that the observed downregulation of branched-chain amino acid (BCAA) biosynthesis and degradation during weaning may be linked to nutritional deficiencies during this period. Additionally, transcriptomic analysis indicated that hub genes before weaning were functionally enriched in pathways associated with protein synthesis and metabolic processes, further suggesting a reduction in protein synthesis and overall growth during weaning. Changes in gut microbiota structure and function also support the presence of nutritional deficiencies in piglets during weaning, as evidenced by a reduction in *Lachnoclostridium* abundance, a genus previously shown to positively correlate with nutrient absorption (Kang et al., 2021). Furthermore, comparative functional analysis of the gut microbiome before and after weaning revealed significant enrichment in metabolic pathways such as 1,4-dihydroxy-6-naphthoate biosynthesis II, 5,6-dimethylbenzimidazole biosynthesis I, L-histidine degradation III, L-glutamate degradation XI, and L-glutamate degradation V before weaning. Previous studies have indicated that the downregulation of these pathways corresponds to an increased demand for energy metabolism (Reiner and Levitz, 2018; Weckmann et al., 2018; Li et al., 2021). Therefore, our results suggest that piglets experience nutritional deficits during the weaning phase.

In alignment with previous research indicating that the abundance of antibiotic resistance genes (ARGs) dynamically fluctuates during lactation in cow manure (Liu et al., 2019), our study observed ARGs changed significantly in Bama miniature piglets during weaning. Specifically, the diversity of ARGs increased after weaning, coinciding with the increased diversity of gut microbiota in the piglets. Greater intestinal microbial diversity implies a higher potential for different types of ARGs, genetic exchange, and horizontal gene transfer, which collectively contribute to the emergence of new ARGs (Pal et al., 2016). Horizontal gene transfer allows resistance genes to spread among strains, thereby facilitating adaptation to environmental changes, dietary shifts, and antibiotic pressures (Thomas and Nielsen, 2005). This process contributes to the rise in ARG prevalence and potential risk level after weaning. Our findings revealed an increased proportion of high-risk ARGs (Ranks I and II) after weaning, with aminoglycoside resistance ARGs being the main contributors to this change. Interaction analyses with gut microbiota further showed that aminoglycoside resistance ARGs exhibited the highest mobility, suggesting that the elevated ARG risk levels after weaning may be closely related to aminoglycoside resistance. Tetracycline resistance, one of the most commonly reported resistance types in pig farms worldwide(Xiao et al., 2016; Munk et al., 2018; Van Boeckel et al., 2019; He et al., 2020), represented 48% of all ARGs in adult and finishing pigs(Wang Y. et al., 2021b). Our study similarly found tetracycline resistance to be the most abundant ARG, with its proportion increasing from 32% before weaning to 42% afterward. Interestingly, correlation analysis identified significant associations between several beneficial bacteria, such as *Lawsonia* and *Bacteroides*, and tetracycline resistance genes. This suggests that dietary changes during weaning may caused re-establishment of intestinal microbial homeostasis, accompanied by the introduction of additional resistance genes. Our study indicates that weaning represents a critical period for the establishment of the future resistance gene profile in Bama miniature pigs, warranting careful attention to dietary and medical interventions during this stage. It is also noteworthy that not all ARGs are transcriptionally active. For example, Wang et al. found through metagenomic and metatranscriptomic analyses that 49.4, 66.5, and 56.6% of ARGs were expressed in the gut microbiota of humans, chickens, and pigs, respectively, with a significant proportion of ARGs remaining transcriptionally inactive (Wang et al., 2020). While our study has extensively explored changes in ARGs within the gut microbiota of Bama miniature pigs during the weaning period, information regarding ARG expression in these bacterial communities is still lacking. Future research should employ transcriptomic analyses to further investigate the functional states and ecological significance of high-risk ARGs in post-weaning environments.

### 4.2 Multi-omics results indicate gut inflammation during weaning

Emerging evidence increasingly suggests that dysbiosis of the gut microbiota is associated with a wide range of diseases, including inflammatory bowel disease (IBD), cardiovascular disease, allergies, diabetes, and obesity (Bownik and Stępniewska, 2016; Koh et al., 2022). Previous studies have demonstrated a decrease in the abundance of genera such as *Megamonas*, *Anaerostipes*, and *Bifidobacterium* in patients with IBD (Arboleya et al., 2016; Yachida et al., 2019; Mager et al., 2020; Parker et al., 2020; Ren et al., 2020). In our study, these bacterial populations similarly decreased after weaning, suggesting the onset of intestinal inflammation. Furthermore, *Lachnoclostridium* and *Acidaminococcus* are known to ferment polysaccharides and amino acids to produce short-chain fatty acids (SCFAs), such as butyrate and acetate, which play crucial roles in promoting the growth of intestinal epithelial cells, enhancing intestinal barrier function, and maintaining the integrity of the intestinal wall (Zhao et al., 2021). In our study, a decline in these beneficial microbes in piglets post-weaning implies potential disruption of intestinal integrity and the occurrence of inflammation. Moreover, our findings indicate that bacterial taxa related to pathogenicity, such as species of the genera *Chlamydia*, *Clostridioides*, *Corynebacterium*, *Clostridium*, and *Pseudomonas*, were enriched in piglets after weaning. Numerous studies have shown that the presence of these genera is typically indicative of infection (Rao and Malani, 2020; Shirley et al., 2023). Additionally, GH94 family enzymes, recognized for their anti-inflammatory and antioxidant properties (Gao et al., 2019), were found to increase in abundance in piglets after weaning, further suggesting an inflammatory response triggered by weaning. Functional enrichment analysis revealed that the superpathway of polyamine biosynthesis III was significantly enriched in piglets post-weaning, a pathway that is known to be involved in microbial adaptation to diverse physiological and environmental stresses (Wagner et al., 2021).

Both transcriptomic and metabolomic analyses support the hypothesis of inflammation occurring in piglets during the weaning period. DEGs upregulated in piglets after weaning were functionally enriched in complex immune and inflammatory biological processes (Figure 4E). One such gene, *MZB1*, is known to suppress inflammation by modulating mitochondrial function or enhancing antibody secretion (Xiong et al., 2019; Zhang et al., 2020; Ricci et al., 2021). The upregulation of *MZB1* expression post-weaning further supports the occurrence of inflammation in piglets. In metabolomic analysis, ectoine was identified as one of the most significantly upregulated metabolites, while taurocholate was significantly downregulated. These metabolites suggest the presence of intestinal inflammation and may also imply adverse effects on the digestive system following weaning (Bownik and Stępniewska, 2016; Koh et al., 2022). Furthermore, the downregulation of taurine and hypotaurine pathways after weaning may be linked to the inflammatory response, as studies have suggested that under certain disease conditions or severe stress, the demand for these compounds increases while their synthesis may be impaired (Singh et al., 2023).

### 4.3 Complex interactions between host-microbes-metabolites during weaning

Correlation analysis based on multi-omics data elucidated intricate interactions among genes, microbes, and metabolites in piglets undergoing weaning. Notably, there were significant correlations identified between the gene *MZB1*, the bacterial genus *Alistipes*, and the metabolite stachydrine. After weaning, the expression of the *MZB1* gene and the concentration of stachydrine were all upregulated, and the relative abundance of *Alistipes* decreased in piglets. The *MZB1* gene encodes the marginal zone B and B1 cell-specific protein, which has been shown to enhance the secretion of IgA immunoglobulin and plays a critical role in mitigating gut inflammation (Xiong et al., 2019). Stachydrine, a metabolite with anti-inflammatory and antioxidant properties (Liao et al., 2023). *Alistipes* are known to decrease when colitis occurs (Parker et al., 2020). Dietary changes associated with weaning stress can trigger changes in the intestinal environment, and reductions in *Alistipes* may impair intestinal epithelial barrier function, leading to elevated levels of intestinal inflammation in pigs. The observed upregulation of the *MZB1* gene represents a response to alleviate this stress. The increased expression of *MZB1* may activate metabolic pathways that facilitate the synthesis of stachydrine, thereby contributing to the reduction of inflammation. This study emphasizes that weaning stress activates a network of genes, microbes, and metabolites, which collectively contribute to an adaptive response to both external and internal changes. However, the precise mechanisms underlying the interactions among *MZB1*, *Alistipes*, and stachydrine remain unknown and warrant further investigation.

Additionally, network analysis identified bacteria of the genus *Dialister* as having the highest degree of connectivity. The abundance of *Dialister* was reduced in piglets after weaning, and it exhibited strong correlations with the downregulation of the 2-AGPE metabolite and decreased expression of the RHBDF1 gene. Previous research by Joossens et al. noted ecological dysregulation and a reduction in the abundance of *Dialister* in patients with Crohn’s disease (Fukui, 2019). The arachidonic acid moiety of 2-AGPE serves as a precursor for the synthesis of a variety of biologically active lipids, including prostaglandins and leukotrienes, which are key mediators of inflammatory and immune responses (Trostchansky et al., 2021). The *RHBDF1* gene has been implicated in the activation of epidermal growth factor receptor (EGFR)-mediated cell growth signals, as well as other activities that are essential for cellular responses under stressful conditions (Ji et al., 2022). Moreover, silencing the *RHBDF1* gene has been found to suppress inflammatory responses (Gao et al., 2021). We propose that interactions among *Dialister*, 2-AGPE, and *RHBDF1* may play a pivotal role in modulating the response to dietary changes and gut inflammation during weaning, though the detailed mechanisms require further elucidation.

## 5 Conclusion

Bama miniature pigs are the most widely used porcine model animals in China. With the multi-omics technology, this study found significant changes in the growth rate, gut microbiota, metabolites, and gene expression in Bama miniature pigs during weaning. Specifically, the reduction in metabolites of lipids and lipophilic molecules, the downregulated abundance of bacteria *Bifidobacterium*, and the increase of microbial carbohydrate enzymes were associated with the digestion of complex fibers, reflecting the dietary changes during weaning. Meanwhile, the beneficial bacteria such as *Bifidobacterium* reduced and bacteria *Chlamydia* associated with infection increased after weaning, indicating the occurrence of intestinal inflammation. The differential expression genes before and after weaning significantly enriched in immune and inflammatory pathways, and the significant changed metabolites, ectoine and taurocholate, were related to inflammation. Both results further support the inflammation in intestine during weaning. We found the composition and abundance of ARGs changed during weaning. Among the ARGs, tetracycline resistance was most abundant, accounting for 32 and 42% of all ARGs before and after weaning, respectively. Combining multi-omics data, we identified significant correlations between gene *MZB1*, genera *Alistipes* and metabolite stachydrine; and correlations between bacteria *Dialister*, 2-AGPE metabolite and *RHBDF1* gene. Our study reveals complex interactions between gut microbes and host in response to the weaning stress in the Bama miniature pigs, providing scientific foundation for the development of health treatment strategies in miniature pig breeding. The results provide preliminary evidence for understanding the interactions between microbes and hosts during the weaning period, laying a foundation for future research with larger sample sizes. However, given the limited sample size of this study, further researches with more individuals and longer time periods are needed to validate and expand upon the findings of this study.

## Data Availability

The original contributions presented in the study are publicly available. This data can be found at: https://www.ncbi.nlm.nih.gov, accession number PRJNA1147800.

## Footnotes

^1^ https://www.ncbi.nlm.nih.gov/

^2^CAZy, http://www.cazy.org/

^3^CARD, https://card.mcmaster.ca/
