# Erratum: Multi-omics analysis reveals interactions between host and microbes in Bama miniature pigs during weaning

**DOI:** 10.3389/fmicb.2025.1556615

**Published:** 2025-01-23

**Authors:** Frontiers Production Office

**Affiliations:** Frontiers Media SA, Lausanne, Switzerland

**Keywords:** weaning, multi-omics, interactions, gut microbes, metabolites, gene expression

Due to a production error, the funder National Natural Science Foundation of China (32171607) was erroneously omitted. The corrected funding statement appears below:

