# Supplementary figures and images for: Multi-omics analysis reveals interactions between host and microbes in Bama miniature pigs during weaning
Source: Front Microbiol. 2024 Dec 11;15:1482925. doi: 10.3389/fmicb.2024.1482925 (PMC11668797; doi:10.3389/fmicb.2024.1482925)

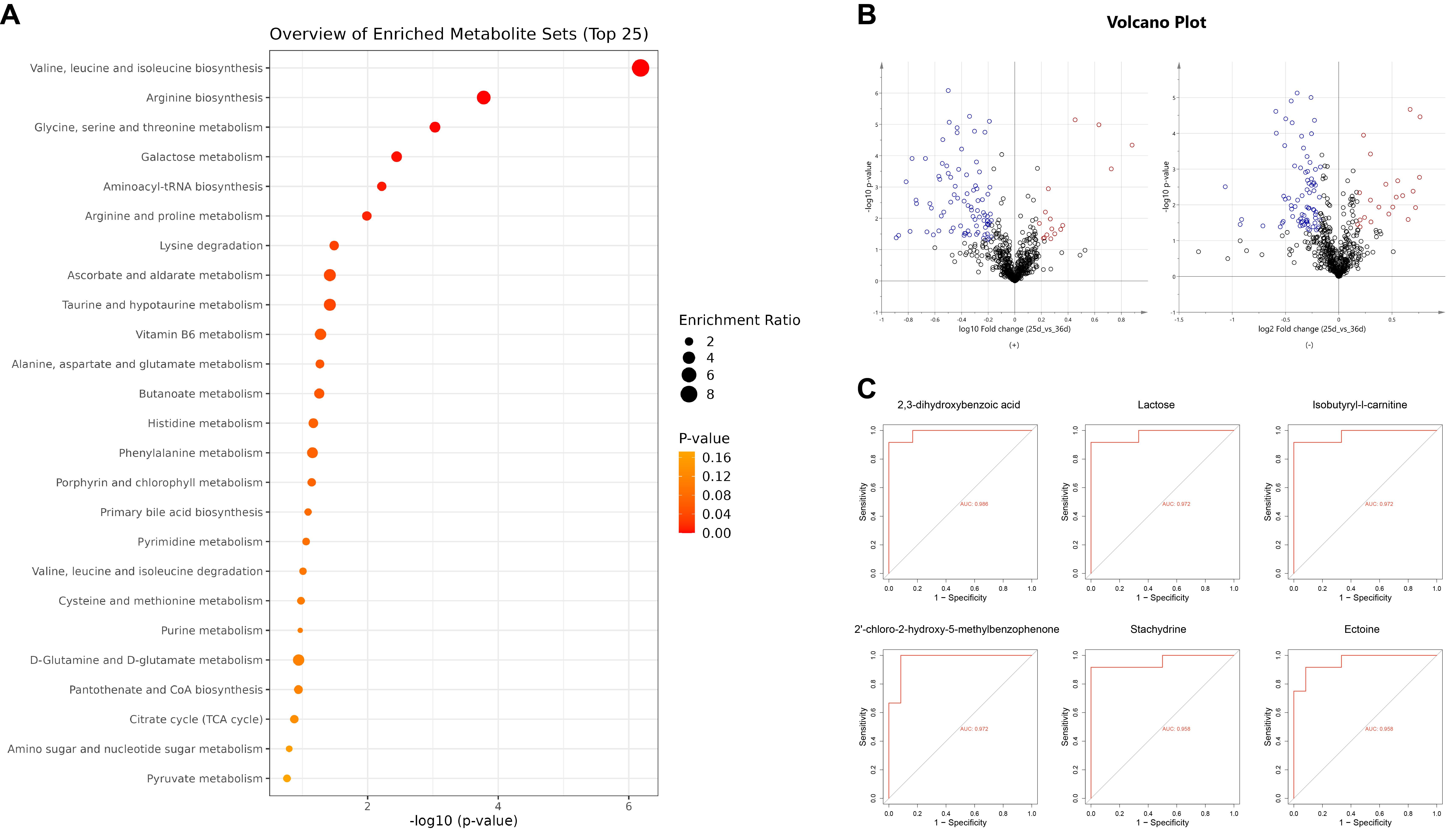

Supplement: Supplementary file 1 [file Image_1.JPEG]

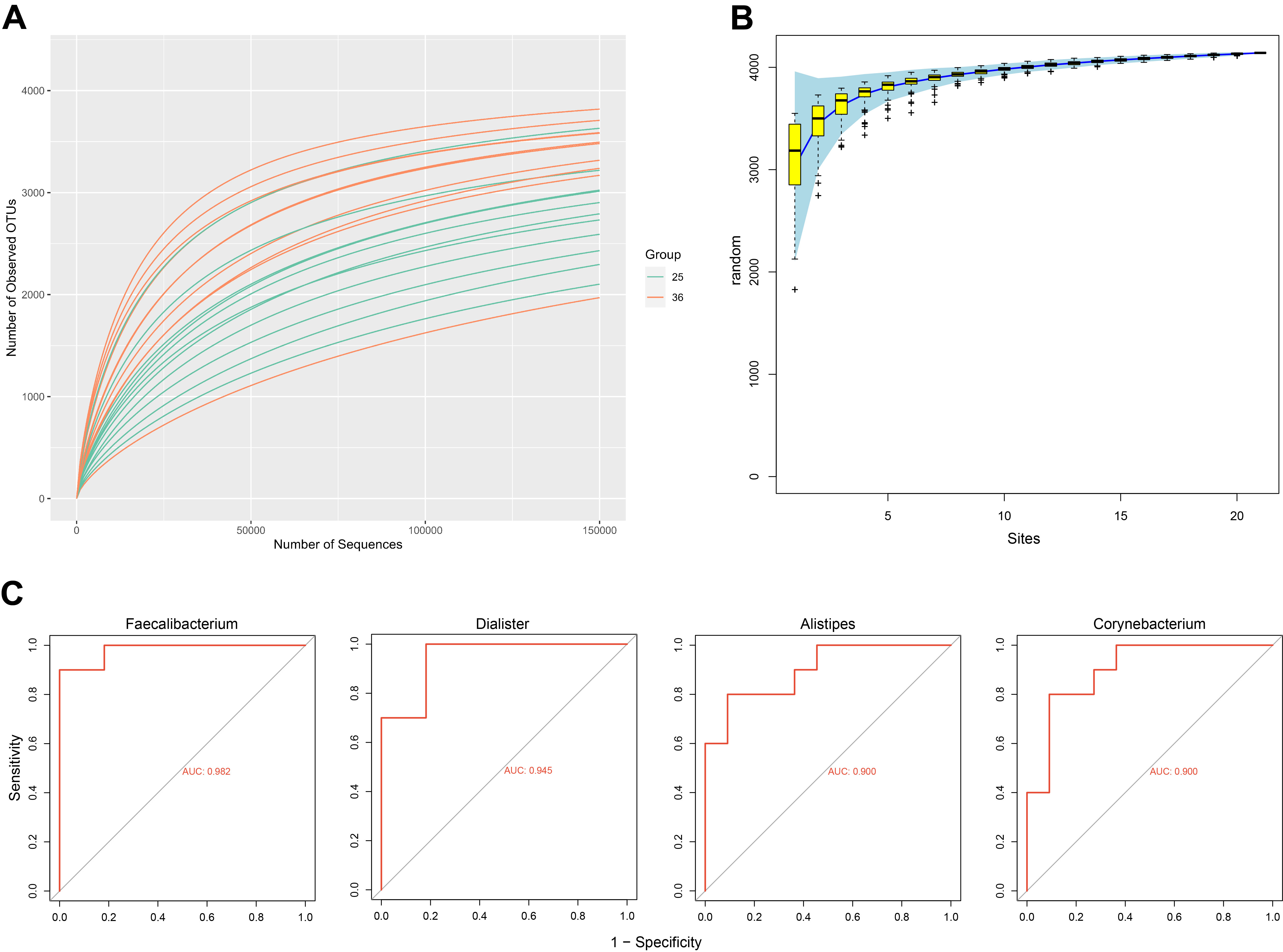

Supplement: Supplementary file 2 [file Image_2.JPEG]

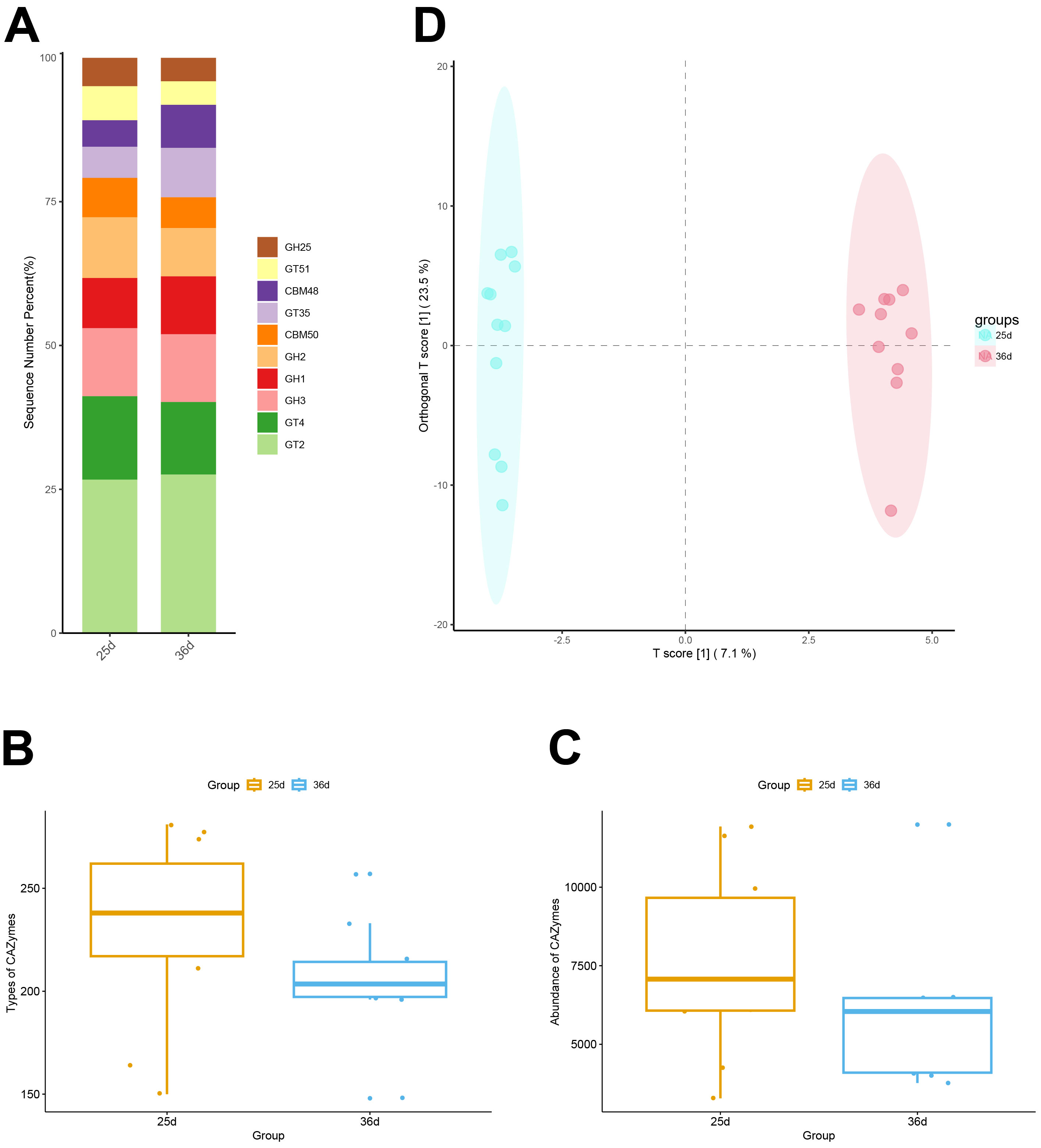

Supplement: Supplementary file 3 [file Image_3.JPEG]

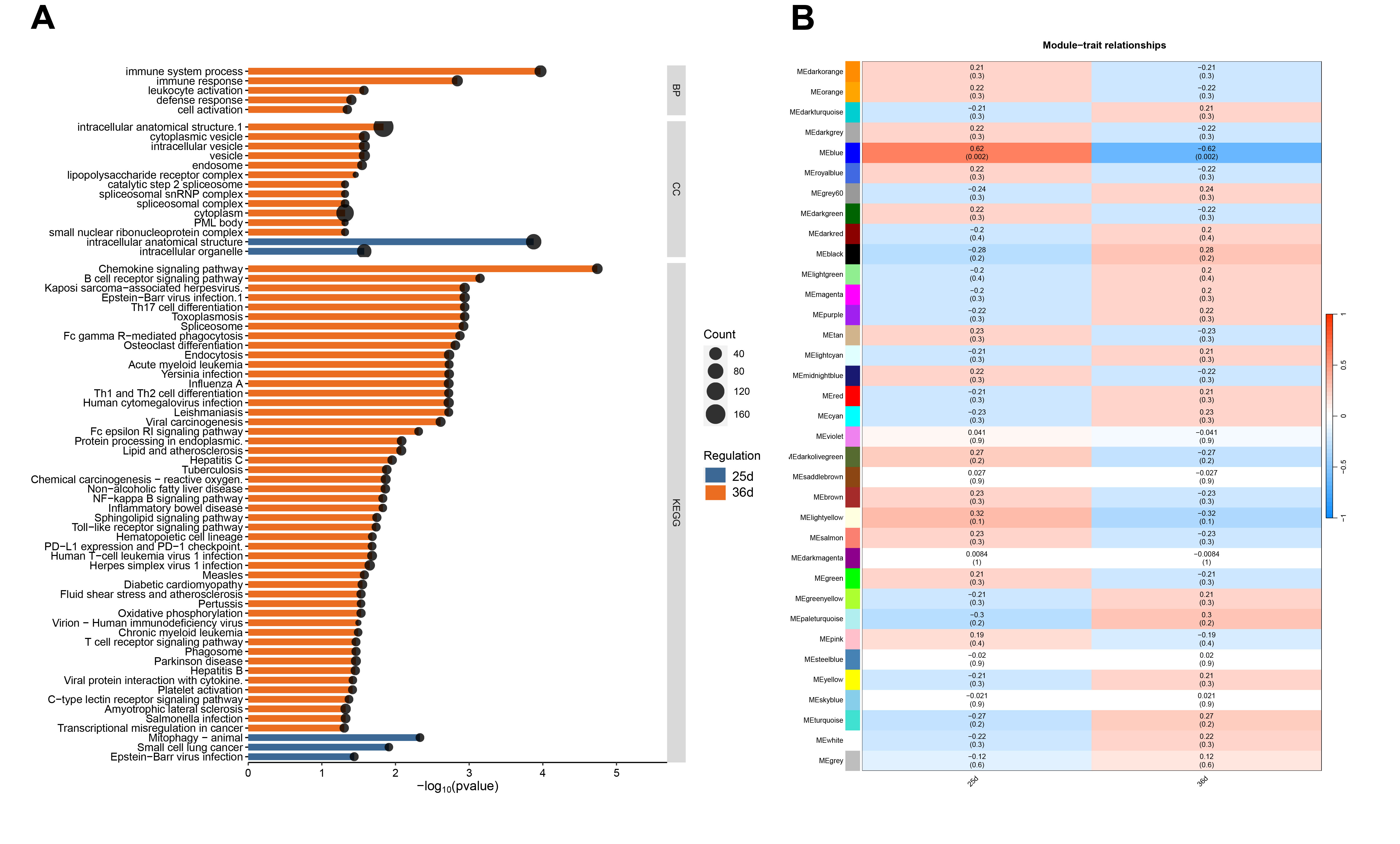

Supplement: Supplementary file 4 [file Image_4.JPEG]
